# Gastric Adenocarcinoma in Helicobacter pylori-Negative Autoimmune Gastritis: A Case Report and Literature Review

**DOI:** 10.7759/cureus.66910

**Published:** 2024-08-15

**Authors:** Hiroshi Kishikawa, Sakiko Takarabe, Masataka Ichikawa, Aya Sasaki, Jiro Nishida

**Affiliations:** 1 Gastroenterology, Tokyo Dental College Ichikawa General Hospital, Chiba, JPN; 2 Clinical Laboratory, Tokyo Dental College Ichikawa General Hospital, Chiba, JPN

**Keywords:** histology, endoscopy, pepsinogen, gastric cancer, helicobacter pylori, autoimmune gastritis

## Abstract

Recent studies have suggested that gastric cancer does not occur in patients with* Helicobacter pylori-*negative autoimmune gastritis (AIG); however, this notion is controversial. We encountered a case of gastric cancer associated with AIG in which *H. pylori *infection was excluded. A woman in her 70s was referred to our hospital for endoscopic resection of an antral adenoma. An *H. pylori* antibodies test, stool antigens test, *H. pylori* culture, and histological analysis using Giemsa staining yielded negative results. AIG was suspected because the antrum was endoscopically normal but the body was severely atrophic, which are typical findings of AIG. Anti-parietal cell antibodies were 40-fold positive, the gastrin level was 2950 pg/ml, and the pepsinogen I level, pepsinogen II level, and pepsinogen I/II ratio were 6.3 ng/ml, 5.7 ng/ml, and 1.1, respectively. A pathological examination of the gastric body revealed severe oxyntic atrophy with hyperplasia of enterochromaffin-like cells, whereas the antrum showed no pyloric gland atrophy or inflammation. These findings indicated that the patient had *H. pylori*-negative AIG. Four years later, a depressed lesion in the lower body and a flat lesion at the angle were observed; the former was a poorly cohesive carcinoma, and the latter was a differentiated adenocarcinoma. Surgical resection revealed that the lesion in the lower body was a poorly cohesive carcinoma invading the submucosa with vascular involvement, whereas the lesion in the angle was an intramucosal differentiated adenocarcinoma. A review of previous studies of gastric cancer with *H. pylori*-negative AIG suggested that patients with histologically and serologically advanced gastritis are at high risk for carcinogenesis. Even in *H. pylori*-negative cases, severe gastric mucosal atrophy in AIG cases may indicate a carcinogenic risk; therefore, surveillance for gastric cancer is especially recommended for these cases. Large cohort studies on the association between *H. pylori*-negative AIG and gastric cancer are warranted.

## Introduction

Autoimmune gastritis (AIG) is associated with an increased risk of gastric cancer and an incidence of 0.5% per person per year [1]. The development of gastric cancer explained by the Correa cascade in the absence of *Helicobacter pylori* infection has been questioned, and some recent studies have suggested that patients with AIG without *H. pylori *infection do not develop gastric cancer [2-4]; however, this idea is controversial [5,6].

Accurately diagnosing AIG cases without *H. pylori *infection is not an easy task. To achieve this, it is necessary to 1) exclude cases with a history of eradication by performing a detailed evaluation, 2) confirm negative *H. pylori * test results using two or more modalities to exclude current cases of *H. pylori *infection, and 3) confirm corpus-restricted histologic gastritis with spared antrum to exclude cases of current and past *H. pylori* infection.

The presence or absence of a current *H. pylori *infection in AIG is difficult to diagnose because severe atrophy can reduce the abundance of *H. pylori*. For cases involving severe atrophy, the urea breath test, stool antigen test [7], histology [8], and serology may provide false-negative results because of the low* H. pylori* abundance in the gastric mucosa. Therefore, two or more diagnostic modalities should be used to avoid false-negative results. In AIG, advanced atrophy may result in the disappearance of *H. pylori*; therefore, a histological evaluation of gastritis using a gastric biopsy specimen is the most reliable diagnostic method because it can exclude current and past *H. pylori *infections [4]. *H. pylori *infection is known to cause histological changes in the gastric mucosa that extend sequentially from the antrum to the gastric body, and this change sometimes extends to the body. In contrast, in patients with AIG without *H. pylori* infection, histological changes induced by autoimmune mechanisms extend sequentially from the gastric body to the distal stomach (i.e., in the opposite direction compared to that associated with *H. pylori* gastritis), and this change does not extend to the antrum. Therefore, histologic findings other than reactive gastropathy are not typically observed in the antrum with *H. pylori*-negative AIG [4]. Considering these findings, histological evidence compatible with AIG in the gastric body, excluding the antrum, strongly suggests the absence of current and past *H. pylori *infections in AIG. We describe a case of gastric cancer associated with *H. pylori*-negative AIG diagnosed based on this strict definition.

## Case presentation

**Figure 1:**
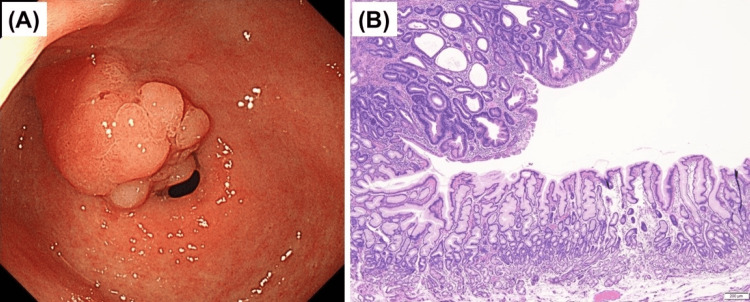
Endoscopic and histologic findings of intestinal-type adenoma in the gastric antrum A) White light endoscopy reveals an elevated round lesion in the gastric antrum adjacent to the pylorus ring. B) Histological findings of the endoscopically resected specimen. The pyloric mucosa surrounding the intestinal-type adenoma with low-grade dysplasia shows no atrophy or intestinal metaplasia with corkscrew-like foveolar hyperplasia, suggesting reactive gastropathy (hematoxylin and eosin staining; magnification, ×40).

**Figure 2:**
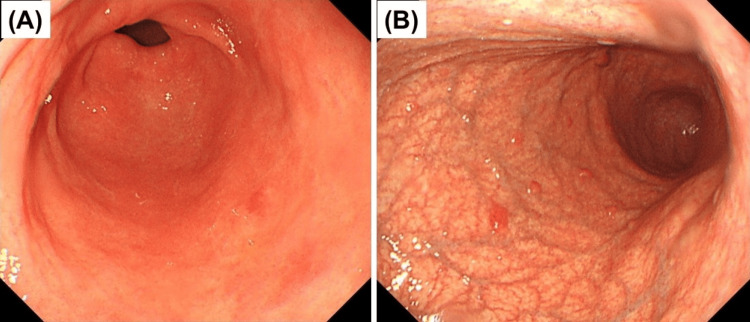
Endoscopic findings of background mucosa suggestive of autoimmune gastritis A) Conventional white light endoscopic findings of the gastric antrum showing no atrophy. B) Conventional white light endoscopic findings of the gastric body showing severe atrophy with marked vascular visibility on the entire great curvature of the gastric body and scattered hyperplastic polyps. Endoscopy of the gastric antrum and body reveals “corpus-dominant advanced atrophy,” which is a typical feature of autoimmune gastritis (AIG).

A woman in her 70s presented to our hospital with an antral adenoma. Upper gastrointestinal endoscopy revealed a flat whitish lesion in the prepylorus (Figure 1A), which was endoscopically resected. Pathological testing of the specimen revealed an adenoma, and the background mucosa exhibited reactive gastropathy; however, pyloric atrophy and intestinal metaplasia were not observed (Figure 1B). We noticed that the gastric mucosa of the antrum was endoscopically normal (Figure 2A); however, the corpus was severely atrophic (Figure 2B) and exhibited “corpus-dominant advanced atrophy,” which led us to suspect AIG. Her anti-parietal cell antibodies were 40-fold positive, anti-intrinsic antibodies were negative, gastrin level was 2950 pg/ml (normal range: 37-172 pg/ml), pepsinogen I level was 6.3 ng/ml, pepsinogen II level was 5.7 ng/ml, pepsinogen I/II ratio was 1.1 (normal pepsinogen level: pepsinogen I >70 pg/ml or pepsinogen I/II ratio >3), and vitamin B12 level was 135 pg/ml (normal range: 233-914 pg/ml).

**Figure 3:**
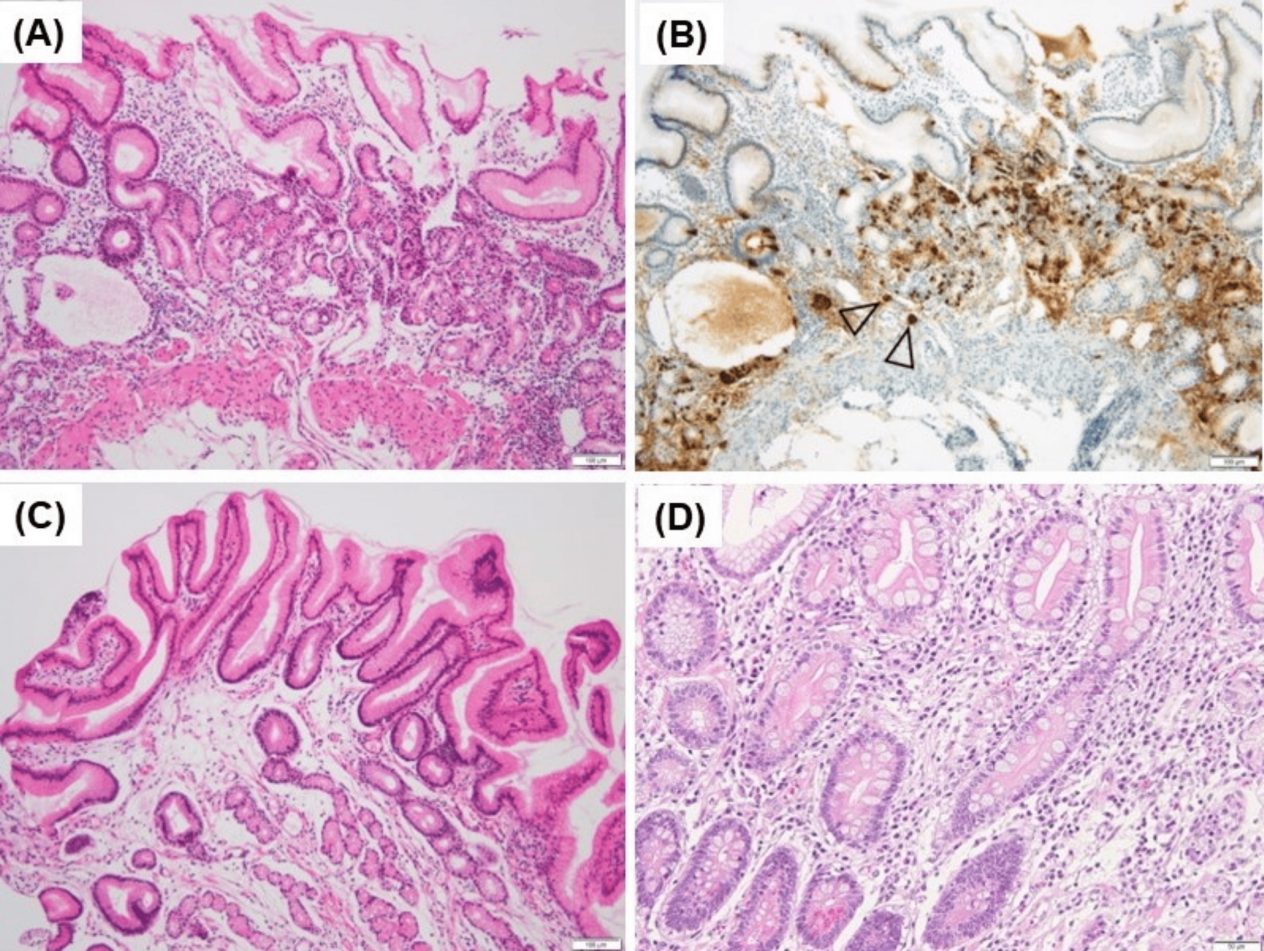
Histological findings of background mucosa compatible with autoimmune gastritis (AIG) without current and past H. pylori infection A) Histological findings of the oxyntic mucosa biopsy reveal a marked reduction in the oxyntic gland, including the absence of parietal cells, intestinal metaplasia, pseudopyloric metaplasia, and thickened muscularis mucosae (hematoxylin and eosin staining; magnification, ×100). B) Immunohistochemical staining with chromogranin A identifies enterochromaffin-like cells that line the long portion of the gastric pit, indicating “linear hyperplasia,” and form small clusters, indicating an endocrine cell micronest (arrowhead) (Chromogranin A staining; magnification, ×100). C) Histological findings of the pyloric mucosa biopsy reveal no pyloric gland atrophy or intestinal metaplasia with foveolar hyperplasia, suggesting reactive gastropathy. Histological findings in the body and antrum are typical of end-stage AIG (hematoxylin and eosin staining; magnification, ×100). D) Complete intestinal metaplasia is recognized in the background mucosa of the corpus (hematoxylin and eosin staining; magnification, ×200).

A biopsy of the greater curvature of the body showed the loss of parietal cells and intestinal metaplasia (Figure 3A). Extraglandular micronodular hyperplasia of enterochromaffin-like cells was observed using chromogranin A staining (Figure 3B). The biopsy of the antrum showed no pyloric gland atrophy and no inflammatory cell infiltration (Figure 3C). Complete intestinal metaplasia was also observed in the background mucosa of the corpus in the resected specimen (Figure 3D). Because these findings were consistent with the end phase of AIG, and because the laboratory data were consistent with AIG, we diagnosed the case as AIG. An *H. pylori *antibodies test, stool test of *H. pylori *antigens, *H. pylori *culture, and *H. pylori* histology obtained using Giemsa staining yielded negative results. These *H. pylori *test results and pathological findings of the antrum without atrophy (Figures 1B, 3C) suggested that current and past *H. pylori *infections were unlikely; therefore, we suspected pure AIG without *H. pylori* infection.

**Figure 4:**
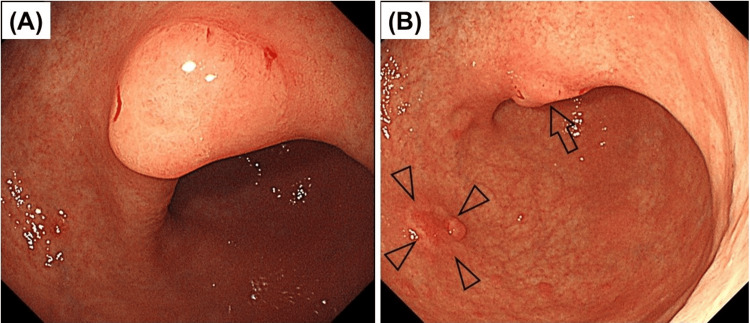
Endoscopic findings of gastric cancers in the angle and body A) Conventional white light endoscopy of the gastric angle. A whitish-raised lesion is observed in the angle. The biopsy results indicate differentiated adenocarcinoma. B) Conventional white light endoscopy of the gastric body. An erythematous depressed lesion with a nodule at the margins is observed in the lower corpus of the greater curvature, where a poorly cohesive carcinoma is detected (arrowhead). At this angle, a whitish-raised lesion (A) is observed (arrow).

**Figure 5:**
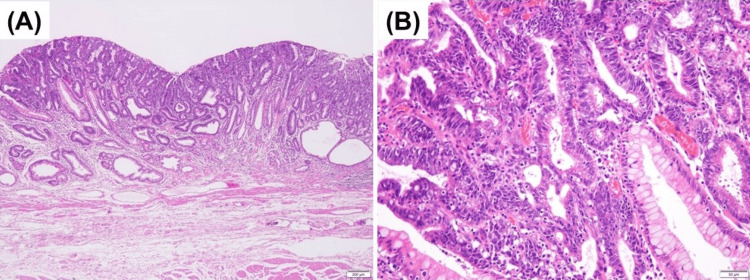
Histological findings of the resected specimen of gastric cancer in the angle A)  Microscopic findings of the resected specimen of the lesion in the gastric angle. Differentiated adenocarcinoma localized in the mucosa without invasion into the submucosal layer is observed (hematoxylin and eosin staining; magnification, ×40). B) Highly magnified image. The right side of the specimen shows a well-differentiated adenocarcinoma with minimal glandular alterations, whereas the left side shows severe glandular alterations (hematoxylin and eosin staining; magnification, ×200).

**Figure 6:**
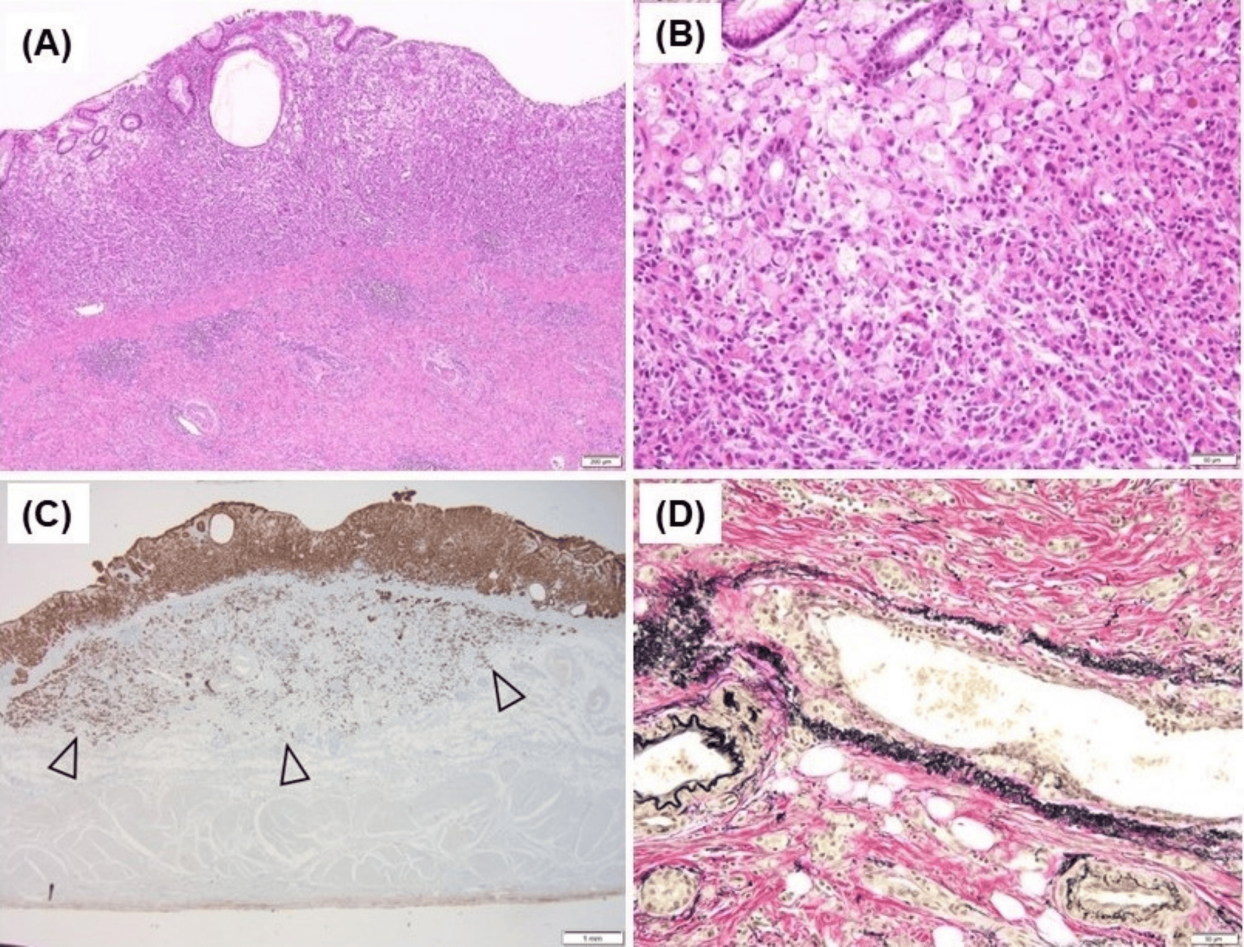
Histological findings of the resected specimen of gastric cancer in the body A) Microscopic findings of the resected specimen of the lesion in the gastric body. Poorly cohesive carcinoma invading the submucosal layer is observed (hematoxylin and eosin staining; magnification, ×40). B) Highly magnified image. Classic signet ring cell carcinoma is observed in the superficial layer. Poorly cohesive carcinoma is observed in the deep layer (hematoxylin and eosin staining; magnification, ×200). C) Cytokeratin AE1/AE3 staining indicates that the tumor has invaded the deeper submucosal layers just above the muscle layer (arrowhead) (Cytokeratin AE1/AE3 staining; magnification, ×12.5). D) Elastica van Gieson staining of the vein reveals intravascular invasion of tumor cells, suggesting positive lymphovascular invasion (Elastica van Gieson staining; magnification, ×200).

Four years later, endoscopy revealed a flat lesion in the lesser curvature of the antrum (Figure 4A) and a depressed lesion in the lower body (Figure 4B); however, a biopsy revealed differentiated adenocarcinoma in the lesser curvature of the antrum and poorly cohesive carcinoma in the lower body. Surgery was performed, and a pathological examination of the resected specimen revealed that the lesion at the gastric angle was an intramucosal differentiated adenocarcinoma (Figures 5A-5B) and that the lesion in the gastric body was a poorly cohesive carcinoma with a signet ring cell component invading the deep layer of the submucosa (>1 mm) with lymphovascular involvement (Figures 6A-6D). According to the 8th edition of the American Joint Committee on Cancer, the final pathological stage was stage I (T1b, N0, M0).

## Discussion

We observed a case of gastric cancer associated with *H. pylori*-negative AIG. A recent study that evaluated *H. pylori*-negative AIG reported that only low-grade intraepithelial neoplasia occurred and no cases of high-grade intraepithelial neoplasia or gastric cancer were observed, suggesting that carcinogenesis is caused by the associated *H. pylori *infection, but not by AIG itself [3]. However, advanced-stage AIG without *H. pylori *infection presents a characteristic milieu of severe hypoacidity and resultant marked hypergastrinemia, both of which have been implicated in gastric carcinogenesis. Ness-Jensen et al. reported that hypergastrinemia increases the risk of gastric adenocarcinoma in humans, especially in the proximal stomach [9], while, hypergastrinemia induced by proton pump inhibitor use and AIG are considered potential causes of the increased incidence of gastric carcinoma in the proximal stomach, especially in younger populations of several Western countries [10]. Regarding the association between hypergastrinemia and adenocarcinoma, it has been postulated that the risk of undifferentiated gastric cancer increases with dedifferentiation of enterochromaffin-like cells and that the risk of differentiated gastric cancer increases with gastric stem cells [11]. Microbe overgrowth in the stomach associated with gastric hypoacidity induces N-nitrosation, which is strongly associated with gastric carcinogenesis [12]. Although there have been numerous studies and meta-analyses of the association between proton pump inhibitor use and the development of gastric cancer [13,14], hypoacidity and hypergastrinemia induced by proton pump inhibitor use are considered the putative causes of carcinogenesis. Taken together, these basic and clinical findings suggest that advanced AIG in patients without *H. pylori* infection may progress to gastric cancer because of the high-risk environment for carcinogenesis caused by hypergastrinemia and hypoacidity that is similar to or worse than that of patients treated with proton pump inhibitors.

Compared to complete intestinal metaplasia, incomplete intestinal metaplasia is associated with a higher carcinogenic risk. Several studies based on the hypothesis that *H. pylori*-negative AIG induces complete rather than incomplete intestinal transformation have reported that *H. pylori*-negative AIG is less likely to induce gastric cancer [4]. However, Angerilli et al. recently reported that complete intestinal metaplasia is not rare in patients with AIG accompanied by gastric cancer (5/26 (19%) cases in their study) [15]. In our case, the background mucosa showed complete intestinal metaplasia. These findings suggest that complete intestinal metaplasia in the background mucosa is a risk factor for carcinogenesis and that the likelihood of gastric carcinogenesis may be higher when carcinogenic environments are present.

During this study, we collected cases of strict *H. pylori*-negative AIG-associated gastric cancer (as in our case). These cases met the following criteria: (1) no history of eradication and two or more* H. pylori t*ests with negative results; (2) either positive anti-parietal cell antibodies or positive anti-intrinsic factor antibodies; and (3) pathological findings of the gastric body compatible with AIG (severe atrophy of oxyntic glands, metaplasia, diffuse lymphoplasmacytic infiltration, enterochromaffin-like cell hyperplasia etc.) without pyloric gland atrophy, metaplasia and inflammatory cell infiltration in the antrum. As a result, five cases, including the present case, were observed in female patients and included in this study (Table 1) [16-19]. Autoantibodies were more than 40-fold positive for anti-parietal cell antibodies in four patients and positive for anti-intrinsic factor antibodies in two patients. The gastrin level was high (>2500 pg/ml) in all patients. Measurements of the pepsinogen levels revealed that the pepsinogen I level was <10 ng/ml in three patients. Vitamin B12 levels of only four patients could be measured. Of these four patients, three had vitamin B12 levels less than 200 pg/ml. Endocrine cell micronests were observed in four of the five patients. Gastric cancers included one case of a hyperplastic polyp that progressed to carcinoma, one case of fundic-type gastric adenocarcinoma, and two cases of differentiated adenocarcinoma. Three cases remained in the mucosa, suggesting that they were compatible with high-grade dysplasia according to the 8th edition of the American Joint Committee on Cancer. Two cases invaded the submucosa. Four lesions were located in the corpus. Two lesions were located in the antrum.

**Table 1:** Gastric cancer accompanied by autoimmune gastritis without current and past H. pylori infections M: mucosal layer; SM: submucosal layer; SAT: stool antigen test; UBT: urea breath test

Case	Author	Age (years)	Sex	Tumor characteristics						Autoantibody		Serological markers				H. pylori test with negative result	Endeocrine cell micronests
				Location	Size	Morphology	Histology	Invasion Depth	Lymphatic vascular invasion	Anti-parietal cell antibody (titer)	Anti-intrinsic factor antibody	Pepsinogen test		Gastrin (pg/mL)	Vitamin B12 (pg/mL)		
												Pepsinogen I (ng/ml)	Pepsinogen I/II ratio				
1	Sumida et al. [16]	87	Female	Corpus	5 mm	Flat	Gastric adenocarcinoma of fundic gland type	M	Negative	Positive (1:40)	Positive	NA	NA	3992	NA	Serology, SAT	NA
				Corpus	7 mm	Protruded	Tubular adenocarcinoma	M									
2	Yamanaka et al. [17]	55	Female	Corpus	20 mm	Protruded (polyp-like)	Tubular adenocarcinoma	SM	Positive	Positive (1:80)	Positive	7.6 ng/ml	<0.5	>3000	208	Serology, SAT	Positive
3	Minaguchi et al. [18]	56	Female	Antrum	32 mm	Depressed	Tubular adenocarcinoma	M	Negative	Positive (1:20)	NA	NA	0.8	5100	151	Serology, UBT	Positive
4	Ozawa et al. [19]	90s	Female	Corpus	27 mm	Flat	Tubular adenocarcinoma	SM	Negative	Positive (1:40)	Negative	5.6 ng/m	0.4	7660	103	Serology, UBT, SAT	Positive
5	Kishikawa et al. (current study)	70s	Female	Corpus	25 mm	Depressed	Poorly cohesive carcinoma	SM	Positive	Positive (1:40)	Negative	6.3 ng/m	1.1	2900	135	Serology, SAT, Culture, Histology	Positive

Because the cutoff values for pepsinogen I, which predicts AIG, and gastrin were approximately 20 ng/ml and 345 ng/ml [20], respectively, low pepsinogen I and high gastrin levels in the reported cases indicated serologically advanced AIG. High gastrin levels may have been caused by preserved G cells attributable to the absence of atrophy in the antrum with *H. pylori*-negative AIG. Most reported cases of *H. pylori*-negative AIG with gastric cancer complications were accompanied by pathological endocrine cell micronests. Endocrine cell micronests arise from intraductal linear enterochromaffin-like cell hyperplasia and are observed during late-stage AIG; therefore, the reported cases of AIG can be interpreted as both serologically and histologically advanced. Most gastric cancers in AIG are thought to be associated with *H. pylori *infection; however, in* H. pylori*-negative AIG, the absence of *H. pylori* carcinogenic risk may require a high degree of gastric mucosal atrophy.

## Conclusions

Our case of *H. pylori*-negative AIG was complicated by both differentiated adenocarcinoma and poorly cohesive carcinoma. Based on previous case reports of gastric cancer in patients with *H. pylori*-negative AIG, advanced-stage AIG may be associated with a high risk of gastric cancer. Therefore, surveillance for gastric cancer in patients with *H. pylori*-negative AIG is particularly necessary, especially for those with advanced atrophy. Discussions of the clinical characteristics of *H. pylori*-negative AIG complicated with gastric cancer and risk factors for gastric cancer have been limited to case reports. Therefore, large cohort studies that include more *H. pylori*-negative AIG cases diagnosed according to an unbiased and strict definition are necessary to confirm these findings.
